# Differential expression of THOC1 and ALY mRNP biogenesis/export factors in human cancers

**DOI:** 10.1186/1471-2407-11-77

**Published:** 2011-02-17

**Authors:** María S Domínguez-Sánchez, Carmen Sáez, Miguel A Japón, Andrés Aguilera, Rosa Luna

**Affiliations:** 1 Centro Andaluz de Biología Molecular y Medicina Regenerativa CABIMER, Universidad de Sevilla-CSIC, Av. Américo Vespucio s/n, 41092 Sevilla, Spain; 2 Instituto de Biomedicina de Sevilla (IBIS) and Department of Pathology, Hospital Universitario Virgen del Rocío/Universidad de Sevilla/CSIC, Av. Manuel Siurot s/n, 41013 Sevilla, Spain

## Abstract

**Background:**

One key step in gene expression is the biogenesis of mRNA ribonucleoparticle complexes (mRNPs). Formation of the mRNP requires the participation of a number of conserved factors such as the THO complex. THO interacts physically and functionally with the Sub2/UAP56 RNA-dependent ATPase, and the Yra1/REF1/ALY RNA-binding protein linking transcription, mRNA export and genome integrity. Given the link between genome instability and cancer, we have performed a comparative analysis of the expression patterns of THOC1, a THO complex subunit, and ALY in tumor samples.

**Methods:**

The mRNA levels were measured by quantitative real-time PCR and hybridization of a tumor tissue cDNA array; and the protein levels and distribution by immunostaining of a custom tissue array containing a set of paraffin-embedded samples of different tumor and normal tissues followed by statistical analysis.

**Results:**

We show that the expression of two mRNP factors, THOC1 and ALY are altered in several tumor tissues. THOC1 mRNA and protein levels are up-regulated in ovarian and lung tumors and down-regulated in those of testis and skin, whereas ALY is altered in a wide variety of tumors. In contrast to THOC1, ALY protein is highly detected in normal proliferative cells, but poorly in high-grade cancers.

**Conclusions:**

These results suggest a differential connection between tumorogenesis and the expression levels of human THO and ALY. This study opens the possibility of defining mRNP biogenesis factors as putative players in cell proliferation that could contribute to tumor development.

## Background

Gene expression involves multiple processes from transcription to mRNA processing, export and translation. During transcription, the nascent pre-mRNA associates with RNA-binding proteins and undergoes a series of processing steps, resulting in export-competent mRNA ribonucleoprotein complexes (mRNPs) that are exported to the cytoplasm [1]. Eukaryotic cells have developed quality control mechanisms that prevent the export of suboptimal mRNPs and synthesis of dysfunctional proteins [2]. Aberrant expression of mRNA binding proteins affect different steps of mRNA metabolism, significantly altering gene expression. The physiological relevance of mRNP biogenesis control is supported by the fact that altered expression or dysfunction of some RNA binding proteins are associated with various diseases including cancer, as for example that of some 3'-end processing factors and of some proteins involved in alternative splicing [3,4].

The THO complex is a conserved eukaryotic nuclear complex that functions in mRNP biogenesis [5]. This complex was first isolated in *Saccharomyces cerevisiae *as a four-protein complex composed of stoichiometric amounts of Tho2, Hpr1, Mft1, and Thp2 [6]. THO has also been purified in *Drosophila *and human cells and the complexes contain counterparts of the yeast subunits Hpr1 and Tho2, called Thoc1 and Thoc2 respectively, as well as additional components such as Thoc5-Thoc7 and hTex1/Thoc3 [7,8]. THO interacts physically and functionally with proteins involved in mRNA export: the Sub2/UAP56 RNA-dependent ATPase, and the Yra1/REF1/ALY RNA-binding protein; forming a larger complex termed TREX (transcription-export complex) [8]. Yeast THO, *sub2 *mutant and to a lesser extent *yra1 *mutants show similar phenotypes of transcription impairment, mRNA export defects and transcription-associated hyperrecombination which indicate that these proteins could act in the same mRNP biogenesis pathway [5,9]. THO and Sub2 can be considered the closest related factors, given the capacity of Sub2 overexpression to suppress THO mutations, and the similarity in the strength of the phenotypes conferred by *hpr1*, *tho2 *and *sub2 *mutations [10,11].

The relevance of THO in cell physiology has been clearly shown from yeast to humans. Yeast THO null mutants are sick and slow growers and THO depletion has a negative effect on growth rate of human and Drosophila cell lines [6,7,12]. Moreover, THO is required for viability of the early mouse embryo and for postnatal survival, as determined by a THOC1 knockout [13]. A connection of THO with cancer development has also been suggested. In human, Thoc1 was identified as a nuclear matrix protein that binds to the retinoblastoma tumor suppressor protein pRb [14]. High levels of hHPR1/THOC1 have been observed in breast and lung cancer cells and are associated with tumor size and aggressiveness [12,15]. However, neither the pattern of expression of THOC1 and other THO components and related proteins in different tumors and the possible mechanism underlying this process are known. Although, data of gene expression derived from microarray and systematic protein localization analyses are available [16,17], little is known about the expression of these genes in a wide range of cancers and its relation with the pathologies of patients. To get further insight into the role of mRNP biogenesis factors in cancer an analysis was performed of the expression pattern of THO and other functionally related factors such as ALY and hSpt4 in different human tumors. The results showed that both the expression of THOC1 and ALY is altered in several tumor tissues, suggesting a connection of these mRNP biogenesis factors with tumorogenesis. A comparative analysis of the expression pattern of these genes using tissue tumor arrays reveals differences between them that could be compatible with a different role in the mRNP biogenesis and relevance in other biological processes.

## Methods

### Materials

cDNA probes were obtained by PCR of cDNA clones purchased from I.M.A.G.E. consortium. Mouse monoclonal anti-THOC1 and anti-ALY were purchased from Abcam, and secondary reagents were purchased from Dako for immunohistochemistry analysis (IHC).

### Cell Lines

Breast cancer cell lines (MCF7, SKBr-3 and T47-D), and MCF10-A were purchased from American Type Culture Collection and propagated according to the conditions recommended by the vendor.

### Tissue microarrays

1 mm tissue cores were obtained from archival paraffin blocks of tumors resected at Hospital Universitario Virgen del Rocío, and then arrayed into recipient paraffin blocks. Pathological diagnoses are provided in Additional file 1. The collection and use of the human material was approved by the local ethics committee.

### Quantitative Real-Time Reverse Trancription-PCR Analysis

Total RNA was isolated from breast cell lines using Purescript RNA isolation kit (Gentra System), cDNA was synthesized with transcriptor first-strand cDNA synthesis kit (Roche) and Real-time PCR was performed with SYBR green dye in the 7500 Real Time PCR system of Applied Biosystems by following the manufacturer's instructions. THO-UAP56 and ALY specific primers were designed using Primer Express software (the sequence is available upon request). The HPRT1 gene was used as an endogenous control after evaluating different housekeeping genes in several breast cancer cell lines and identifying HPRT as the most stable internal control.

### Immunohistochemistry for THOC1 and ALY

Immunohistochemical analyses (IHC) were performed on tissue microarray four-micron sections. Monoclonal antibodies against THOC1 and ALY were used. Tissues were microwaved in 1 mM EDTA buffer (for ALY) or treated with 4N HCl and trypsin for THOC1. Primary antibody incubation was overnight at 4°C. Secondary reagents (Dako) were applied according to manufacturer's protocol. Slides were then counterstained with hematoxylin and mounted in DPX. Sections where the primary antibody was omitted were used as negative controls. Immunostaining was evaluated at least on 10 microscopic fields at × 200 by two independent pathologists and unequivocal immunostained cells were counted. Immunohistochemical expression was scored as strong (>50% of the carcinoma cells stained), weak (10%-50%) or negative (<10%). Digital images were acquired using a Zeiss microscope.

### Statistical analysis

A two-tailed paired t-test was used to determine whether the differences in THOC1, ALY and hSPT4 cDNA levels in tumor versus normal samples were statistically significant. To determine the association between the expression levels of ALY with a high grade tumor, a chi-squared test was performed.

## Results

### Deregulation of THOC1 and ALY mRNA Expression in Tumors

**Figure 1:**
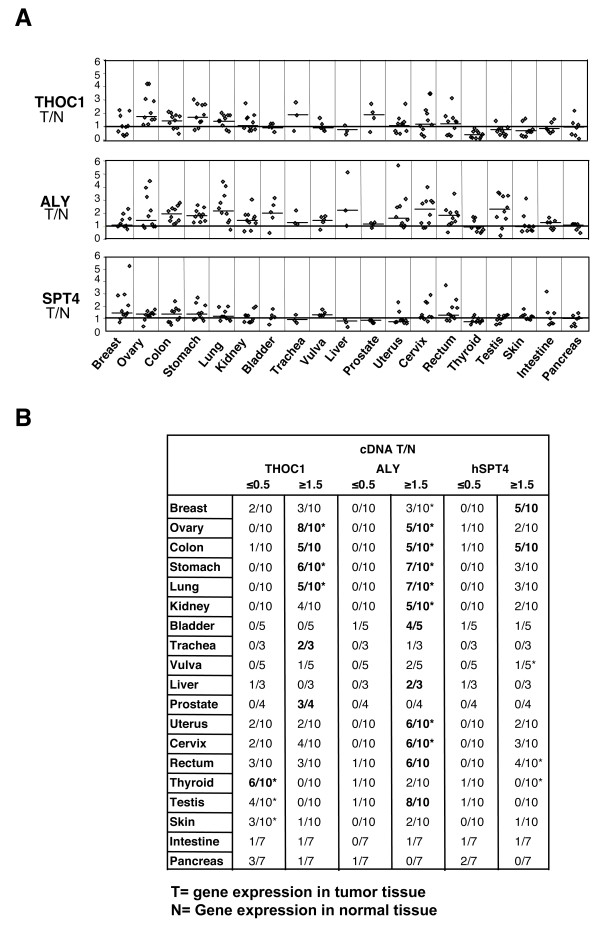
**THOC1, ALY and hSPT4 expression in paired normal/tumor tissues by cDNA array**. **(A) **A Cancer Profiling Array II (Clontech), representing cDNA pools from matched tumor and normal tissue, was hybridized with specific [^32^P]dCTP-labeled THOC1, ALY and hSPT4 probes, according to manufacturer's instructions. cDNA expression values were calculated by quantification of signal intensities in a FUJI FLA 3000. Gene expression in paired tumor/normal tissues from the same patient was quantified considering expression in normal tissues as one unit. Upper panel represents the ratio tumor/normal of each pair in the different types of tumors. N, normal; T, tumor. **(B) **Number of tumors showing upregulation (T/N > 1.5) or downregulation (T/N < 0.5) of THOC1, ALY and hSPT4 genes. The number is marked in bold in that tissues where the genes were up- or down-regulated in ≥50% of the tumors analyzed. A two-tailed paired t-test was used to determine whether the differences in cDNA levels in tumor versus normal samples were statistically significant (marked with *, for details see Additional file 3).

First the mRNA levels of THOC1 and ALY in tumor tissues by hybridization of a Cancer Profiling Array II (Clontech) were evaluated. THOC1, was taken as representative of factors that act at the transcription level such as THO-UAP56, and ALY, as a related factor that serves as adaptor in mRNA export. We also analyzed the expression of hSpt4, the subunit of the human transcription elongation factor DSIF, [18], which participates early in transcription and is not related to mRNA export. The array contains normalized cDNAs from tumor and matched normal tissues from a variety of organs (154 cDNAs pair samples derived from 19 different tissues and 3 to 11 patients by tissue). The results of the hybridization and the relative expression in tumor samples calculated as the ratio of tumor versus normal signal (T/N) are shown in Figure 1A and Additional file 2. We considered those cases in which the values of T/N ratio of cDNAs were outside the interval 0.5-1.5 (Figure 1B). THOC1 was ubiquitously expressed in all normal tissues (Additional file 2) and a significant increase was found in ovary, stomach and lung tumors (marked with *; for more details see Additional file 3) with the highest levels of overexpression in ovary (4 fold higher than those of the matched normal samples) (Figure 1A). In addition, a statistically significant reduction of THOC1 transcripts was observed in thyroid, testis and skin tumors, even though in the later the differences were lower (Figure 1). We have compared our results with the SAGE (Serial Analyses of Gene Expression) data of THOC1 available at the human Gene Compendium GeneCard http://www.genecards.org. The GeneCard data show changes in THOC1 mRNA levels in tumor versus normal tissues, in almost all samples analyzed: higher levels in lymphoid node, breast, lung, colon, liver, pancreas, prostate and skin and lower in thyroid, cortex and cerebellum. In contrast our analyses report significant changes in only a subset of the tissues analyzed, as indicated above.

The expression pattern of ALY was analyzed with the same cDNA array. Hybridization with an ALY specific probe revealed a general and strong signal for ALY transcripts in all normal tissues tested, with the highest levels in testis (Additional file 2). ALY mRNA was increased in a wide variety of tumors, whereas it was not significantly reduced in any tissue (Figure 1, Additional file 3). SAGE analyses show also that ALY mRNA levels are higher in a number of tissues, except in thyroid and cortex tissues in which a strong reduction is observed.

Expression of the transcription factor hSPT4 in tumors was similar to those of normal matched tissues in almost all the different organs studied (except vulva, rectum and thyroid).

From the analyses of cDNA arrays we can conclude that there is a differential pattern of expression of THOC1 and ALY in tumor tissues. However, given the number of comparisons made (see Additional file 3), we cannot discard that in some cases the comparison might reach the chosen level of significance by random. Therefore, the analyses of cDNA arrays indicate that THOC1 is differentially expressed in some tumors, both upregulated and downregulated, whereas ALY is overexpressed in a broad range of tumors and only a few significant changes were observed for hSPT4.

### Up-regulation of THO-UAP56 and ALY expression in breast cancer cells

**Figure 2:**
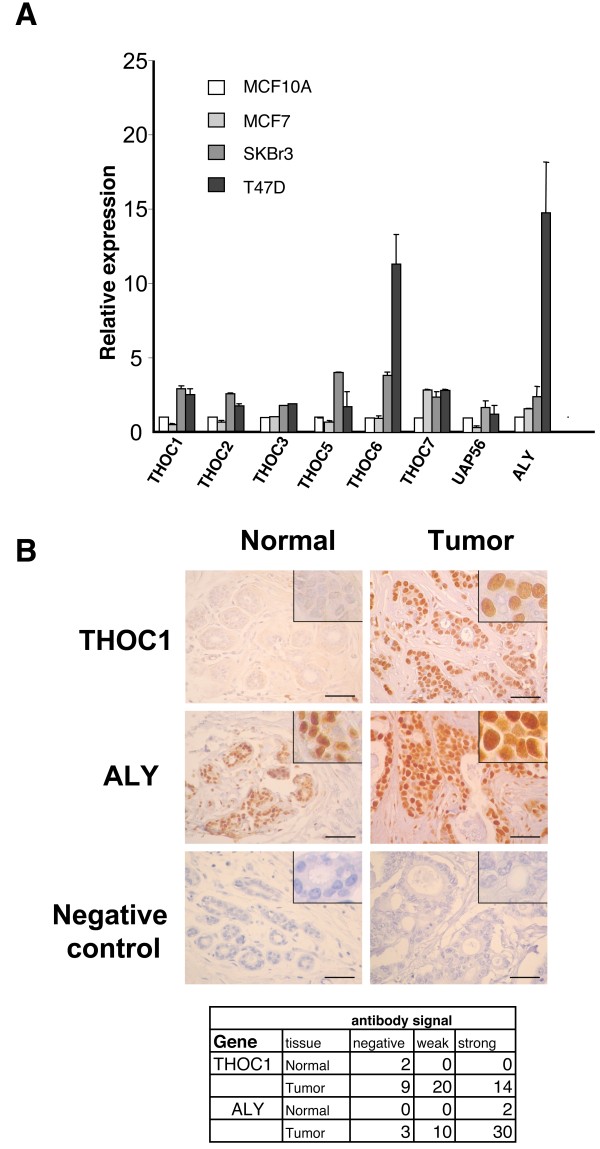
**Expression of THO-UAP56 and ALY in breast tumor cell lines**. **(A) **mRNA levels of THOC1, THOC2, THOC3, THOC5, THOC7, UAP56 and ALY were determined by real-time quantitative PCR (RT-PCR) in breast tumor cell lines MCF7, SKBr3 and T47-D and compared to those of the non tumor cell line MCF10-A. Data are representative of two independent experiments, and quantitative PCRs were repeated three times for each one. Errors bars show standard deviation (SD). **(B) **Immunohistochemical analysis of THOC1 and ALY expression in normal breast tissues and breast carcinomas. Immunohistochemical analysis was performed on formalin-fixed, paraffin-embedded specimens (see Table below). After incubation with antibodies the slides were counterstained with hematoxylin. Bars represent 50 μm. Expression levels of THOC1 and ALY were determined in a semi-quantitative way (negative staining; weak staining and strong nuclear positivity, for more details see Materials and Methods). In the upper panel a representative photograph of normal and tumor tissue is shown. At the bottom negative controls with no primary antibody are included. Inserts show details at higher magnification. The results of the immunohistochemical analysis of 43 samples are summarized in the table below.

It has been described that THOC1 is overexpressed in breast cancer [12]; however, we only detected 3 out of 10 tumor samples with mRNA levels higher than the corresponding normal samples. We decided to study in more detail the expression of THO and other mRNP factors in breast tissues. We evaluated the expression of the components of the human THO complex, THOC1, THOC2, THOC3, THOC5, THOC6 and THOC7, as well as the expression levels of UAP56 and ALY in breast cancer cell lines (Figure 2). mRNA levels were analyzed by real-time-PCR in the malignant breast tumor cell lines MCF7, SKBr3 and T47-D and compared to those of the breast immortalized cell line MCF10-A. An increase in the expression of all components of the THO complex was observed in the breast cancer cell lines, the expression being stronger in SKBr3 and T47D, the most aggressive of the breast tumor cell lines analyzed (Figure 2A). Interestingly, THOC6 mRNA levels were the highest. Although preliminary, these data could indicate that in some cases the stoichiometry of THO is altered and suggest that THOC6 could play additional roles apart from the THO complex [19,20]. RT-PCR of UAP56 and ALY indicated that these genes were also overexpressed in breast cancer cell lines. The study was extended by performing immunostaining analysis of THOC1 and ALY in normal and tumor breast tissues in order to see if the protein levels of these factors had also increased. Specific antibodies were used to immunostain an array containing 43 breast cancer samples and normal breast controls. A weak signal for THOC1 protein was detected in the cytoplasm of normal ductal epithelial cells and a strong nuclear signal in 80% of the tumor samples analyzed (Figure 2B). Immunohistochemical analysis using anti- ALY revealed a strong expression of this protein in the nucleus of epithelial glands in normal breast tissues. This nuclear signal was observed with at least the same intensity in 70% of the breast tumor samples analyzed (Figure 2B). Nevertheless, given the high expression levels of the ALY protein the sensitivity of this array to detect an increase in ALY expression is limited. These data are in agreement with the overexpression of hHPR1/THOC1 previously reported in breast cancer cell lines [12] and could indicate an up-regulation of the expression of ALY in breast cancer, at least at the mRNA level.

### Comparative analysis of THOC1 and ALY proteins using a tumor tissue array

**Figure 3:**
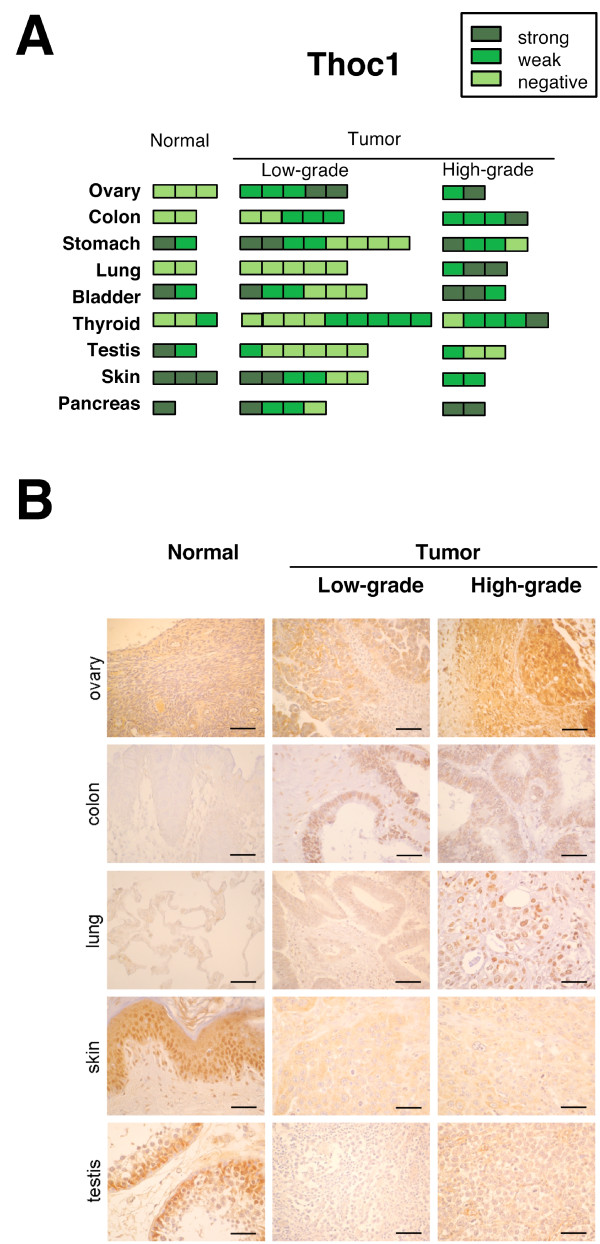
**Immunohistochemical analysis of THOC1 in normal and tumor tissues**. A series of tumors and normal samples from different tissues was tested by immunohistochemistry using surgical paraffin-embedded samples. Tumors were classified as low-grade and high-grade (pathological diagnoses are detailed in Additional file 1). **(A) **Results of the semi-quantitative analysis of the inmunohistochemistry of THOC1 are shown. **(B) **Figures correspond to representative inmunohistochemical analysis of normal and tumor samples. Bars represent 50 μm. For other details see Figure 2. THOC1 is weakly expressed in the cytoplasm and nuclei of normal ovarian, colon and lung, and it is intensively expressed in the nucleus of ovarian, colon and lung carcinoma samples. THOC1 protein levels are reduced in skin and testis tumors: a strong signal is detected in the epidermis of normal skin and germ cells of normal testis, whereas skin tumors and seminomas show a negative or weaker cytoplasmic staining.

**Figure 4:**
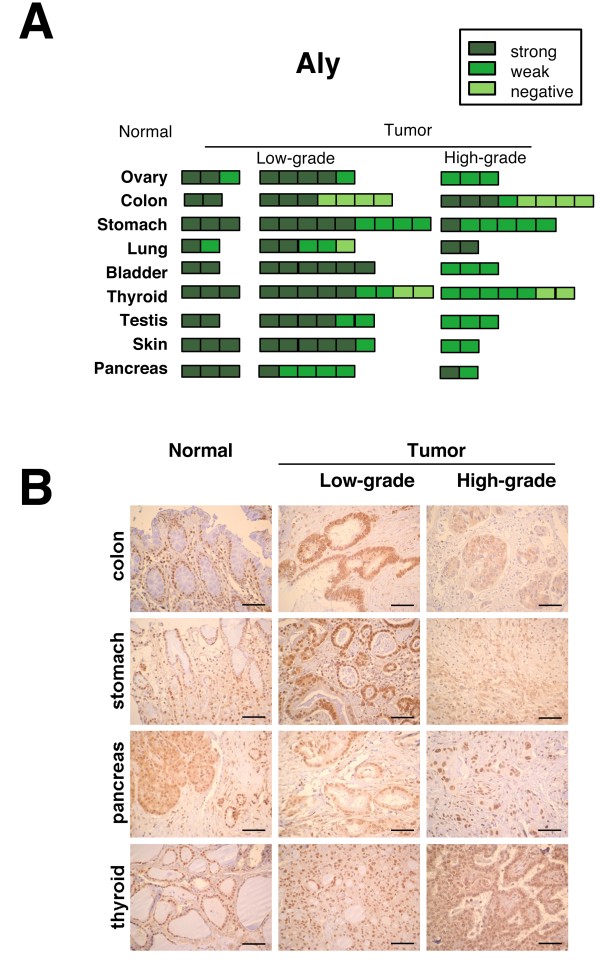
**Immunohistochemical analysis of ALY in normal and tumor tissues**. **(A) **Results of the semi-quantitative analysis of the inmunohistochemistry of ALY are shown. **(B) **Figures correspond to representative inmunohistochemical analysis of normal and tumor samples. Bars represent 50 μm. For other details see Figure 3. ALY protein levels are intensively detected in the nuclei of normal and low-grade tumors of colon, stomach, thyroid and pancreas, whereas some of more advanced tumors such as colon and stomach present a decrease of the nuclear staining.

Next, we decided to extend the comparative analysis between THOC1 and ALY, with the analysis by immunostaining of the protein levels and distribution of these factors in tumor samples (Figure 3 and Figure 4). For this purpose, a custom tissue array containing a set of paraffin-embedded samples of different tumor and normal tissues was created. The array contained a representation of tumor specimens of those organs in which, according to the results of the analysis of the cDNA tumor array, THOC1 and ALY could be expected to be deregulated. It was done with a total of 121 samples including approximately 3 normal tissue controls and an average of 9 samples covering low and high-grade tumors for each organ analyzed (for more details see Additional file 1). As can be seen in Figure 3, THOC1 staining revealed that the gene was expressed in all normal tissues with the highest expression in skin and pancreas. The lower signal was detected in stomach, bladder and testis, and the weakest staining was observed in ovary, colon, lung and thyroid. An increase in THOC1 protein signal in the nucleus was detected in ovarian, colon and lung tumors (Figure 3A and 3B). Our results suggest that THOC1 could be relevant in ovarian tumors, in which THOC1 expression levels were high in almost all the carcinomas analyzed (Figure 3A and Figure 1). The increase in the expression of THOC1 protein has been confirmed by western blot in some ovarian and lung normal and tumor samples (Additional file 4). A loss of THOC1 protein staining was observed in testis and skin cancers, in up to 75% of the samples. In these tumors the nuclear signal disappeared and a low immunoreactivity was detected in the cytoplasm (Figure 3). In general, the protein data, although IHC is not a real quantitative method, is in agreement with the results obtained by hybridization of the cDNA array in all these tissues, except for stomach and thyroid tumors that showed a protein signal very similar to that of normal samples.

Tissue microarrays provide the possibility to immunohistochemically stain a large number and variety of normal and cancer tissues, as it is the case of the human protein atlas [17]. We have compared our IHC results with those obtained in the human protein atlas. In the case of THOC1 the comparison does not provide reliable results, because there are two protein atlas IHC analyses (HPA019687 and HPA019096) with different conclusions due to the different protein levels detected in the tissues samples. Whereas in the first few cases of breast cancer, malignant lymphomas and melanomas were considered moderately positive, in the second, this was only the case in few cases of lung, stomach, pancreatic and testicular tumors. On the other hand, the THOC1 overexpression that we report in breast and lung tumors is in agreement with the protein atlas data and other previous studies [12,15]. In contrast to the atlas IHC data, we observe THOC1 overexpression in ovarian tumors and a decrease in THOC1 expression in testis and skin tumor tissues (Figure 3 and Figure 1). Altogether, these results strengthen the importance of additional studies to establish a reliable association with tumorogenicity and THO expression.

Immunohistochemical analyses were performed with specific antibody anti-ALY, (Figure 4), a strong nuclear signal for the ALY protein being detected in almost all the normal tissues, in contrast to the tissue differential THOC1 immunostaining.

Interestingly, ALY signal was shown to have an equal intensity in half of the tumor samples as in normal tissues (in 46 out of 94 tumors) (Figure 4A and Table 1). The intensity of the immunostaining with anti-ALY was reduced in colon, stomach, thyroid, testis and pancreas tumor samples with a cytoplasmic and lower signal than the control samples in almost 50% of the tumors analyzed (Figure 4B).

**Table 1:** Inmunohistochemical analysis of ALY protein

samples which staining intensity is weaker than normal/total tumor samples specified			
**TUMORS**	**all tumors**	**low-grade tumors**	**high-grade tumors**
**ovary**	0/8	0/5	0/3
**colon**	9/15	4/7	5/8
**stomach**	9/15	4/9	5/6
**lung**	1/7	1/5	0/2
**bladder**	3/9	0/6	3/3
**thyroid**	11/16	4/9	7/7
**testis**	5/9	2/6	3/3
**skin**	3/8	1/6	2/2
**pancreas**	5/7	4/5	1/2
**TOTAL NUMBER**	**46/94**	**20/58**	**26/36**

Comparison of ALY staining in low-grade and high-grade tumor populations. Association of high-grade tumors and weaker staining of ALY protein was analysed by χ2 test ( P value = 0.0004).

When we compare our ALY IHC array with those of the protein atlas IHC data (reports CAB016281 and HPA019799, which show close to 90% of coincidence), it becomes evident that whereas the Protein Atlas shows that ALY is highly expressed in normal tissues and in a wide range of tumors, in our study half of the tumors showed a lower overall signal and an additional cytoplasmic distribution (Figure 4A and Table 1).

### The ALY protein is poorly detected in high-grade tumors, but highly detected in normal proliferative cells

A priori, the reduction of the ALY protein contrasts with the up-regulation of ALY mRNA observed after hybridization of the cDNA array (Figure 1). However, when the ALY protein signal was analyzed in the different tumors, it was clear that a significant reduction of ALY protein levels occurs only in the high-grade tumors (a weak or negative staining in 35% of low-grade tumors -20 out of 58- versus 72% -26 out of 36- in high-grade tumors) (Figure 4A and Table 1). This datum suggests that ALY protein levels are down-regulated in the most dedifferentiated phases of the tumor. The ALY protein atlas IHC data shows that only a small proportion of tumor samples (13-23%) had a lower signal than normal tissues and do not provide sufficient data to make conclusions on the relationship between ALY expression and tumor grade.

**Figure 5:**
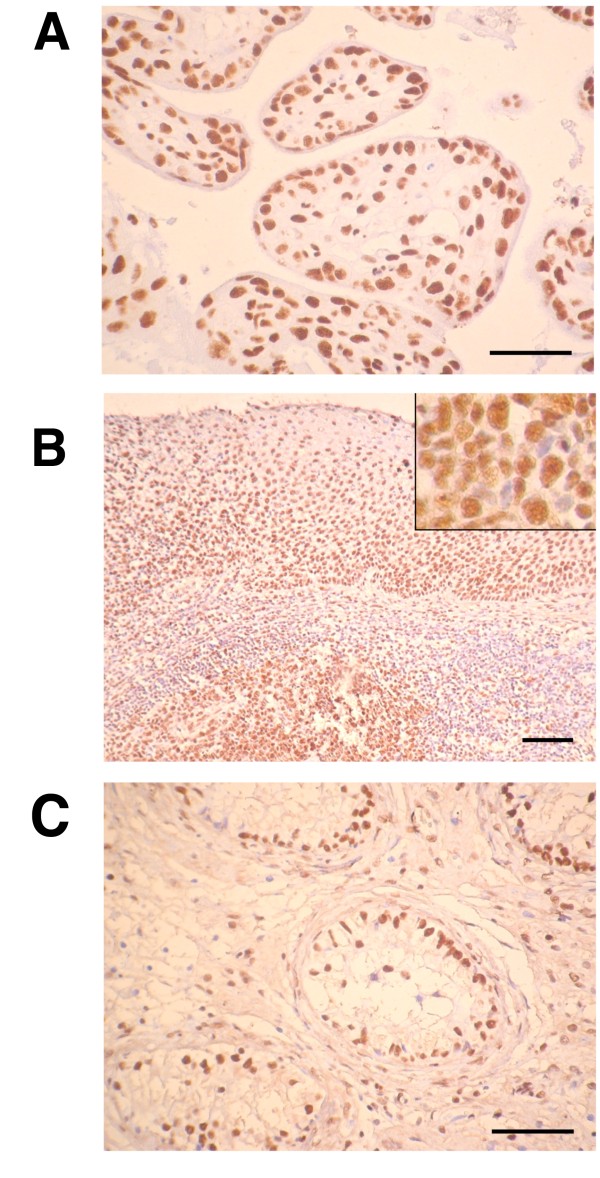
**Detection of ALY in normal proliferative cells**. Strong immunostaining of **(A) **trophoblast of normal placenta **(B) **epithelium and lymphoid follicles of normal tonsil, with details of follicle center cells (insert) **(C) **germ cells of normal testis. Bars represent 50 μm (A,C) and 100 μm (B).

To gain further insight into the physiological relevance of this reduction, ALY expression was analyzed in different normal tissues with high cell proliferation rate or cells that differentiate into a wide variety of cell types. As is shown in Figure 5, a strong nuclear signal corresponding to the ALY protein was detected in cells of the trophoblast in a normal placenta, in the basal epithelium and lymphoid follicles of normal tonsil and in the germinal cells of testis. A positive signal was observed in dividing cells consistent with the high expression observed in the cDNA array.

## Discussion

Our results indicate that disturbed THOC1 and ALY expression are associated with tumorogenesis. Overexpression of THOC1 has previously been correlated to the grade of invasiveness in breast tumor, and also a relationship between the levels of this protein in non-small lung cancers has been suggested [12,15]. This study shows that not only the expression of THOC1, but also that of THO/TREX complex is upregulated in breast cancer cells. Moreover, new links between the expression of THOC1 and different tumors were found. THOC1 mRNA and protein levels are up-regulated in ovarian, and lung tumors and down-regulated in those of testis and skin. Interestingly, we show that ALY, another mRNP biogenesis factor is highly detected in proliferative cells and overexpressed in a broad range of tumors. The different pattern of expression of THOC1 and ALY in tumors could indicate a different relevance for each factor in tumorogenesis. Nevertheless, since many genes are differentially expressed in cancer tissues and cancerous cell populations are highly heterogeneous, any intent of associating a specific expression profile with tumorogenesis needs further studies and confirmations with additional tumor banks [16].

Our data indicates that overexpression of THOC1 and ALY is specifically associated with tumors, and not a consequence of high metabolism and proliferation of tumor cells, since an altered pattern of expression of transcription factors, such as hSpt4 in the cDNA tumor array, was not observed. At the same time, the different expression pattern of THOC1, as representative of THO, and ALY in normal and tumor tissues, indicates a different relevance of these factors in the tumorogenisis process. In fact, altered expression or dysfunction of other RNA binding proteins involved in different pre-mRNA metabolic steps such as splicing, 3'-end formation are implicated in various diseases including cancer, supporting the physiological relevance of mRNP biogenesis control [3]. High levels of the THOC1 protein were observed in the nuclei of ovarian, and lung cancer tumors in comparison with normal tissues. As a reduction of THOC1 was also associated with some cancers such as those of skin and testis, these results argue against the idea that THOC1 expression would be required for proliferation and survival of oncogene-transformed cells [21]. As THO has a role in mRNP biogenesis, likely general among eukaryotes, it is possible that changes in THOC1 expression could affect mRNP formation as previously reported in yeast [9,22]. However, we cannot exclude that the pattern of expression of THOC1 in some tumors could also be related to a specific physiological role of THO in different tissues. It is not clear yet whether the physiological relevance is the same in the different tissues and at different stages of development and differentiation. In this sense, it has recently been shown that THOC1 conditional knockout mice affects testis development [23], and that THOC5 function is critical in bone marrow and hematopoiesis [24]. Therefore THOC1 expression levels might potentially be used as a prognostic indicator in specific tumors, but not a general biomarker.

We provide evidence that high expression levels of ALY are connected to normal proliferative tissues and with a wide variety of tumors. This is in agreement with the proposed role of ALY in cell cycle progression and proliferation [25]. It has been suggested that ALY could be a physiological target of nuclear PI3K signaling, which regulates ALY's subnuclear residency in speckles, as well as, cell proliferation and mRNA export activities through nuclear Akt phosphorylation and phosphoinositide association [25]. Despite this link between mRNA export function and proliferation, other activities of ALY could contribute to tumorogenesis. Thus, ALY has also been proposed as a transcriptional coactivator important for c-myc expression in virally induced leukemias and lymphomas [26], and the yeast homolog Yra1 has been shown to be required for S phase entry [27].

It is particularly interesting that high ALY mRNA levels are found in tumor tissues (see cDNA arrays results), whereas low ALY protein levels are associated with high-grade tumors. It is possible that a post-transcriptional regulation of ALY protein occurs in advanced steps of tumorogenesis. Indeed, ALY is regulated by the AKT kinase, and inhibition of ALY phosphorylation substantially decreases cell proliferation and mRNA export [25]. Interestingly, other proteins involved in mRNA export, such as the yeast THOC1 ortholog Hpr1, have been shown to be a target of ubiquitination [28] and phosphorylation [29].

Interestingly, the expression profile of ALY seems to be similar to that of other related mRNP-export factors, such as UAP56/Sub2 and NXF1/Mex67, which interact physically and functionally with THO. Human protein atlas IHCs for these factors (CAB034012 and CAB016327) show that normal tissues display a strong nuclear signal and tumor cells exhibited moderate to strong nuclear immunoreactivity. Instead, approximately 40% and 65% of the tumor samples showed a lower staining than the normal tissues in the UAP56 and NXF1 IHC reports respectively. The significant association between altered ALY expression and tumors open the possibility that ALY could be considered as a possible tumor prognostic marker.

We can conclude that an alteration in THOC1 and ALY expression is associated with tumorogenesis. Given the small size of samples available it is possible that for some tests a sizeable true effect may not have been detected in this study, which could explain some of the apparent inconsistencies with other studies. However, the different patterns of expression of the proteins in tumors could indicate a different relevance of each factor in tumorogenesis. Importantly, however, their different pattern of expression would be consistent with a different functional role of these proteins in mRNP biogenesis and export. Despite the physical and functional interactions between these factors, THO behaves as a stable structural core in eukaryotic cells [5]. In fact, Sub2/UAP56 and Yra1/ALY have not been found or are only detected in substoichiometric amounts in chromatography purifications of the THO complex under high salt conditions from yeast, Drosophila and human cell extracts [6,7,12]. ALY is not stably associated with THO, and this interaction is mediated by UAP56 and THOC5 [30,19]. Moreover, although ALY/Yra1 recruitment to chromatin was first shown to be dependent on THO and UAP56/Sub2 factors in yeast [31], other factors such as the cap-binding protein CBP80 and the transcription elongation factor Spt6 also contribute to the cotranscriptional loading of ALY protein [32,33]. It seems, therefore, that Yra1/ALY could serve as a bridge between RNA-binding proteins early during mRNP biogenesis, acting upstream during transcription and downstream at mRNA export. The different pattern of expression of THO and ALY in tumors is consistent with a differentiated role of both factors in gene expression, for the correct proliferation of the cell and developmental changes that could take place in tumors.

## Conclusion

These results suggest a differential connection between tumorogenesis and the expression levels of human THO and ALY mRNP biogenesis factors. Our study opens the possibility of defining mRNP biogenesis factors as putative players in cell proliferation and differentiation that could contribute to tumor development.

## Conflicts of Interest Statement

The authors declare that they have no competing interests.

## Authors' contributions

MSD-S carried out Quantitative Real-Time Reverse Trancription-PCR Analysis, cDNA hybridization and data analysis. MSD-S and CS performed the immunohistochemical analysis. CS and MAJ were involved in the sample acquisition, sample selection and pathological diagnosis. RL and AA have coordinated the study, interpretation of data and have prepared the manuscript. All authors reviewed and commented on successive drafts of the manuscript and approved the final manuscript.

## Pre-publication history

The pre-publication history for this paper can be accessed here:

http://www.biomedcentral.com/1471-2407/11/77/prepub

## Supplementary Material

Additional file 1 **Brief description of the pathological diagnoses from the tumor samples included in the tissue array**. The table shows a brief description of the pathological diagnoses. Click here for file

Additional file 2 **Results of hybridization of the cDNA array**. The figure shows the results of hybridization of the cDNA array. THOC1 and ALY are ubiquitously expressed in all normal tissues. The highest THOC1 mRNA levels are observed in thyroid and liver and the lowest in stomach and ovary. ALY probe revealed a similar expression in all normal tissues, with the highest levels in testis. Click here for file

Additional file 3 **Expression of THOC1, ALY and SPT4 determined by hybridization of a Cancer Profiling array with THOC1, ALY and SPT4 probes**. In the table, it is shown the average of intensity (LAU/mm2) found for the THOC1, ALY or SPT4 hybridization probes in normal tissues (N) or tumor tissues (T) and its corresponding statistical error mean (St. error mean). Results were analyzed by using the two-tailed t-test which compares two paired groups by means of calculating the difference between each set of pairs, and which is based on the assumption that the differences in the entire population follow a Gaussian distribution. Samples marked in grey were found to be statistically significant (p < 0.05). Click here for file

Additional file 4 **THOC1 expression in Ovary and Lung tumors**. A) Western blot of THOC1 and β actin (as endogenous control) in ovary and lung tissues. B) THOC1 mRNA relative expression in ovary and lung tissues measured by RT-PCR. Click here for file
