# Supplementary material for: Differential expression of THOC1 and ALY mRNP biogenesis/export factors in human cancers
Source: BMC Cancer. 2011 Feb 17;11:77. doi: 10.1186/1471-2407-11-77 (PMC3050854; doi:10.1186/1471-2407-11-77)
Supplement: Additional file 1 — Brief description of the pathological diagnoses from the tumor samples included in the tissue array. The table shows a brief description of the pathological diagnoses. [file 1471-2407-11-77-S1.PDF]

Additional file 1:
Brief description of the pathological diagnoses from the tumor samples included in the tissue array**.**

**Sample no.**	**Site**	**Diagnosis**
1	Ovary	Normal tissue control
2	Ovary	Normal tissue control
3	Ovary	Normal tissue control
4	Ovary	Endometrioid cystadenocarcinoma, moderately differentiated
5	Ovary	Mixed clear cell and serous adenocarcinoma
6	Ovary	Papillary serous adenocarcinoma
7	Ovary	Endometrioid adenocarcinoma, moderately differentiated
8	Ovary	Endometrioid adenocarcinoma, moderately differentiated
9	Ovary	Endometrioid adenocarcinoma, poorly differentiated
10	Ovary	Mucinous adenocarcinoma, poorly differentiated
11	Ovary	Adenocarcinoma, poorly differentiated
12	Colon	Normal tissue control
13	Colon	Normal tissue control
14	Colon	Normal tissue control
15	Colon	Adenocarcinoma, well differentiated
16	Colon	Adenocarcinoma, well differentiated
17	Colon	Adenocarcinoma, well differentiated
18	Colon	Adenocarcinoma, well differentiated
19	Colon	Adenocarcinoma, moderately differentiated
20	Colon	Adenocarcinoma, moderately differentiated
21	Colon	Adenocarcinoma, moderately differentiated
22	Colon	Adenocarcinoma, moderately differentiated
23	Colon	Adenocarcinoma, moderately differentiated
24	Colon	Adenocarcinoma, poorly differentiated
25	Colon	Adenocarcinoma, poorly differentiated
26	Colon	Adenocarcinoma, poorly differentiated
27	Colon	Adenocarcinoma, poorly differentiated
28	Colon	Adenocarcinoma, poorly differentiated
29	Colon	Adenocarcinoma, poorly differentiated
30	Stomach	Normal tissue control
31	Stomach	Normal tissue control
32	Stomach	Normal tissue control
33	Stomach	Adenocarcinoma, well differentiated
34	Stomach	Adenocarcinoma, well differentiated
35	Stomach	Adenocarcinoma, well differentiated
36	Stomach	Adenocarcinoma, intestinal type, moderately differentiated
37	Stomach	Mucinous adenocarcinoma, moderately differentiated
38	Stomach	Adenocarcinoma, intestinal type, moderately differentiated
39	Stomach	Adenocarcinoma, moderately differentiated
40	Stomach	Adenocarcinoma, moderately differentiated
41	Stomach	Adenocarcinoma, moderately differentiated
42	Stomach	Adenocarcinoma, poorly differentiated
43	Stomach	Adenocarcinoma, poorly differentiated
44	Stomach	Adenocarcinoma, poorly differentiated
45	Stomach	Mucinous adenocarcinoma, poorly differentiated
46	Stomach	Adenocarcinoma, poorly differentiated
47	Stomach	Adenocarcinoma, poorly differentiated
48	Lung	Normal tissue control
49	Lung	Normal tissue control
50	Lung	Normal tissue control
51	Lung	Adenocarcinoma, well differentiated
52	Lung	Bronchioloalveolar adenocarcinoma, well differentiated
53	Lung	Papillary adenocarcinoma, moderately differentiated
54	Lung	Adenocarcinoma, moderately differentiated

55	Lung	Adenocarcinoma, poorly differentiated
56	Lung	Adenocarcinoma, poorly differentiated
57	Lung	Adenocarcinoma, poorly differentiated
58	Bladder	Normal tissue control
59	Bladder	Normal tissue control
60	Bladder	Normal tissue control
61	Bladder	Transitional cell carcinoma, low grade
62	Bladder	Transitional cell carcinoma, low grade
63	Bladder	Transitional cell carcinoma, low grade
64	Bladder	Transitional cell carcinoma, low grade
65	Bladder	Transitional cell carcinoma, low grade
66	Bladder	Transitional cell carcinoma, low grade
67	Bladder	Transitional cell carcinoma, high grade
68	Bladder	Transitional cell carcinoma, high grade
69	Bladder	Transitional cell carcinoma, high grade
70	Thyroid	Normal tissue control
71	Thyroid	Normal tissue control
72	Thyroid	Normal tissue control
73	Thyroid	Follicular adenocarcinoma
74	Thyroid	Follicular adenocarcinoma
75	Thyroid	Follicular adenocarcinoma
76	Thyroid	Follicular adenocarcinoma
77	Thyroid	Papillary adenocarcinoma
78	Thyroid	Papillary adenocarcinoma
79	Thyroid	Papillary adenocarcinoma
80	Thyroid	Papillary adenocarcinoma
81	Thyroid	Papillary adenocarcinoma, follicular variant
82	Thyroid	Papillary adenocarcinoma
83	Thyroid	Follicular carcinoma with anaplasia
84	Thyroid	Follicular carcinoma, oncocytic variant
85	Thyroid	Follicular carcinoma, oncocytic variant
86	Thyroid	Papillary adenocarcinoma
87	Thyroid	Papillary adenocarcinoma, tall-cell variant
88	Thyroid	Papillary adenocarcinoma, tall-cell variant
89	Testis	Normal tissue control
90	Testis	Normal tissue control
91	Testis	Normal tissue control
92	Testis	Classical seminoma
93	Testis	Classical seminoma with necrosis
94	Testis	Classical seminoma
95	Testis	Classical seminoma
96	Testis	Classical seminoma, multinodular
97	Testis	Clasical seminoma with syncytiotrophoblast cells
98	Testis	Atypical seminoma
99	Testis	Atypical seminoma
100	Testis	Mixed malignant germ cell tumor
101	Skin	Normal tissue control
102	Skin	Normal tissue control
103	Skin	Normal tissue control
104	Skin	Squamous cell carcinoma, well differentiated
105	Skin	Squamous cell carcinoma, well differentiated
106	Skin	Squamous cell carcinoma, keratinizing type, well differentiated
107	Skin	Squamous cell carcinoma, moderately differentiated
108	Skin	Squamous cell carcinoma, moderately differentiated
109	Skin	Squamous cell carcinoma, keratinizing type, moderately differentiated
110	Skin	Squamous cell carcinoma, poorly differentiated
111	Skin	Squamous cell carcinoma, poorly differentiated
112	Pancreas	Normal tissue control
113	Pancreas	Normal tissue control

114	Pancreas	Normal tissue control
115	Pancreas	Ductal adenocarcinoma, moderately differentiated
116	Pancreas	Ductal adenocarcinoma, moderately differentiated
117	Pancreas	Ductal adenocarcinoma, moderately differentiated
118	Pancreas	Ductal adenocarcinoma, moderately differentiated
119	Pancreas	Ductal adenocarcinoma, moderately differentiated
120	Pancreas	Ductal adenocarcinoma, poorly differentiated
121	Pancreas	Ductal adenocarcinoma, poorly differentiated
