# Supplementary material for: Differential expression of THOC1 and ALY mRNP biogenesis/export factors in human cancers
Source: BMC Cancer. 2011 Feb 17;11:77. doi: 10.1186/1471-2407-11-77 (PMC3050854; doi:10.1186/1471-2407-11-77)
Supplement: Additional file 2 — Results of hybridization of the cDNA array. The figure shows the results of hybridization of the cDNA array. THOC1 and ALY are ubiquitously expressed in all normal tissues. The highest THOC1 mRNA levels are observed in thyroid and liver and the lowest in stomach and ovary. ALY probe revealed a similar expression in all normal tissues, with the highest levels in testis. [file 1471-2407-11-77-S2.PDF]

THOC1

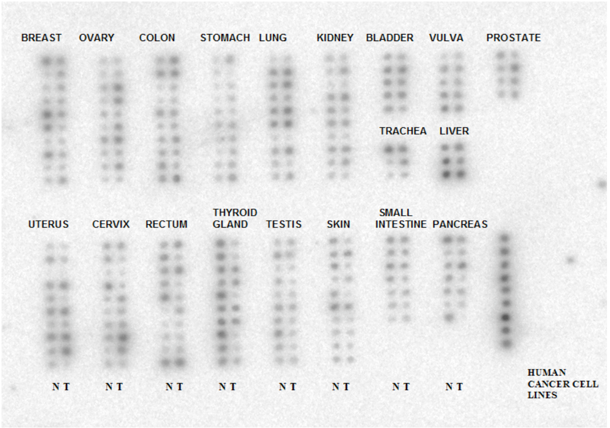

ALY

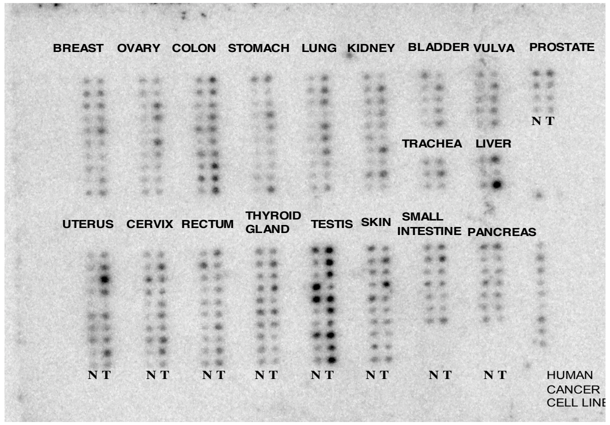

Additional file 2: Results of hybridization of the cDNA array. THOC1 and ALY are ubiquitously expressed in all normal tissues. The highest THOC1 mRNA levels are observed in thyroid and liver and the lowest in stomach and ovary. ALY probe revealed a similar expression in all normal tissues, with the highest levels in testis.
