# Supplementary material for: Differential expression of THOC1 and ALY mRNP biogenesis/export factors in human cancers
Source: BMC Cancer. 2011 Feb 17;11:77. doi: 10.1186/1471-2407-11-77 (PMC3050854; doi:10.1186/1471-2407-11-77)
Supplement: Additional file 3 — Expression of THOC1, ALY and SPT4 determined by hybridization of a Cancer Profiling array with THOC1, ALY and SPT4 probes. In the table, it is shown the average of intensity (LAU/mm2) found for the THOC1, ALY or SPT4 hybridization probes in normal tissues (N) or tumor tissues (T) and its corresponding statistical error mean (St. error mean). Results were analyzed by using the two-tailed t-test which compares two paired groups by means of calculating the difference between each set of pairs, and which is based on the assumption that the differences in the entire population follow a Gaussian distribution. Samples marked in grey were found to be statistically significant (p < 0.05). [file 1471-2407-11-77-S3.PDF]

| | THOC1 | | | | | ALY | | | | | SPT4 | | | | |
|---|---|---|---|---|---|---|---|---|---|---|---|---|---|---|---|
| | N | Sterror mean | T | Sterror mean | p | N | Sterror mean | T | Sterror mean | p | N | Sterror mean | T | Sterror mean | p |
| **Breast** | **19235.1** | 5160.4 | **13513.9** | 1720.1 | **0.239** | **25923.1** | 2222.2 | **33840.4** | 3968.4 | **0.044** | **54196.8** | 5231.0 | **106768.7** | 24530.8 | **0.054** |
| **Ovary** | **8143.3** | 1111.5 | **19047.9** | 3516.1 | **0.005** | **17089.3** | 1063.8 | **32916.2** | 5682.7 | **0.033** | **70781.5** | 8206.2 | **82390.4** | 11337.4 | **0.318** |
| **Colon** | **13675.8** | 2218.2 | **17781.6** | 3186.9 | **0.155** | **33337.1** | 3377.7 | **59630.4** | 4038.2 | **0.000** | **46659.7** | 5453.4 | **52607.5** | 4929.0 | **0.495** |
| **Stomach** | **5261.4** | 735.2 | **9325.5** | 1410.3 | **0.007** | **14264.1** | 2256.1 | **29461.9** | 4536.1 | **0.008** | **45902.2** | 6232.3 | **63181.7** | 7679.2 | **0.064** |
| **Lung** | **14513.5** | 2660.1 | **22759.9** | 5075.1 | **0.013** | **15920.9** | 1248.2 | **40547.8** | 4762.2 | **0.000** | **55416.6** | 5888.5 | **66584.8** | 8233.0 | **0.155** |
| **Kidney** | **10196.4** | 1559.4 | **12668.4** | 2226.6 | **0.193** | **24194.0** | 1987.6 | **36161.4** | 5120.9 | **0.038** | **88386.4** | 8230.8 | **94786.3** | 11125.5 | **0.573** |
| **Bladder** | **20133.0** | 2581.7 | **20604.3** | 4399.5 | **0.859** | **21351.0** | 5670.8 | **33258.6** | 4137.7 | **0.251** | **139398.5** | 48541.7 | **127187.0** | 14413.3 | **0.766** |
| **Trachea** | **20319.0** | 14315.7 | **21109.3** | 7323.7 | **0.937** | **23554.2** | 2983.0 | **37031.7** | 9718.3 | **0.249** | **57380.5** | 17572.9 | **48472.5** | 12537.4 | **0.420** |
| **Vulva** | **15849.0** | 4002.0 | **13826.3** | 1929.6 | **0.409** | **29713.7** | 2518.6 | **40262.5** | 3306.2 | **0.114** | **94728.1** | 8233.8 | **132632.4** | 14134.3 | **0.016** |
| **Liver** | **65242.7** | 21643.8 | **42207.1** | 12860.0 | **0.211** | **32798.1** | 2712.2 | **95614.4** | 43789.5 | **0.274** | **141358.7** | 18279.0 | **84519.8** | 14853.3 | **0.228** |
| **Prostate** | **11431.7** | 1968.3 | **18573.4** | 5177.6 | **0.303** | **27509.5** | 5447.1 | **30954.7** | 6611.5 | **0.319** | **89122.9** | 6250.7 | **124686.2** | 18250.2 | **0.065** |
| **Uterus** | **15159.6** | 2799.4 | **19141.0** | 5344.2 | **0.288** | **29882.1** | 3294.5 | **59390.3** | 11645.7 | **0.037** | **74942.0** | 4017.9 | **70477.2** | 8406.4 | **0.691** |
| **Cervix** | **12127.8** | 2019.7 | **19246.1** | 6217.3 | **0.256** | **23608.4** | 4105.8 | **41354.2** | 4513.0 | **0.008** | **81636.8** | 8106.7 | **117332.3** | 17662.0 | **0.066** |
| **Rectum** | **15904.0** | 3290.9 | **15093.3** | 3575.9 | **0.830** | **23333.5** | 4180.6 | **34851.4** | 2938.9 | **0.060** | **58411.2** | 5782.1 | **88010.6** | 12247.8 | **0.042** |
| **Thyroid** | **39698.7** | 4360.8 | **13455.3** | 2381.3 | **0.001** | **36157.2** | 2475.1 | **35312.5** | 4950.6 | **0.867** | **147452.6** | 12860.9 | **121488.8** | 10734.7 | **0.031** |
| **Testis** | **11770.6** | 1889.4 | **6807.1** | 797.5 | **0.023** | **69243.6** | 16080.0 | **109277.8** | 10662.6 | **0.088** | **207106.6** | 21863.1 | **210172.0** | 28341.1 | **0.853** |
| **Skin** | **11787.7** | 2324.3 | **7291.4** | 1704.6 | **0.050** | **39192.0** | 3171.7 | **43037.3** | 7110.2 | **0.645** | **152237.2** | 10975.3 | **171814.0** | 12037.5 | **0.083** |
| **Intestine** | **12261.9** | 2396.5 | **9543.7** | 1544.0 | **0.106** | **32326.0** | 2108.4 | **37703.1** | 5967.0 | **0.297** | **77435.8** | 13273.1 | **80712.6** | 16785.5 | **0.862** |
| **Pancreas** | **16817.7** | 4320.9 | **12107.1** | 3818.6 | **0.346** | **30821.2** | 3317.4 | **29862.7** | 4406.9 | **0.766** | **115165.8** | 11696.1 | **93863.9** | 18775.1 | **0.346** |

**Additional file 3 Expression of THOC1, ALY and SPT4 determined by hybridization of a Cancer Profiling array with THOC1, ALY and SPT4 probes.** In the table, it is shown the average of intensity (LAU/mm2) found for the THOC1, ALY or SPT4 hybridization probes in normal tissues (N) or tumor tissues (T) and its corresponding statistical error mean (St. error mean). Results were analyzed by using the two-tailed t-test which compares two paired groups by means of calculating the difference between each set of pairs, and which is based on the assumption that the differences in the entire population follow a Gaussian distribution. Samples marked in grey were found to be statistically significant ($p < 0.05$).
