# Supplementary material for: Differential expression of THOC1 and ALY mRNP biogenesis/export factors in human cancers
Source: BMC Cancer. 2011 Feb 17;11:77. doi: 10.1186/1471-2407-11-77 (PMC3050854; doi:10.1186/1471-2407-11-77)
Supplement: Additional file 4 — THOC1 expression in Ovary and Lung tumors. A) Western blot of THOC1 and β actin (as endogenous control) in ovary and lung tissues. B) THOC1 mRNA relative expression in ovary and lung tissues measured by RT-PCR. [file 1471-2407-11-77-S4.PDF]

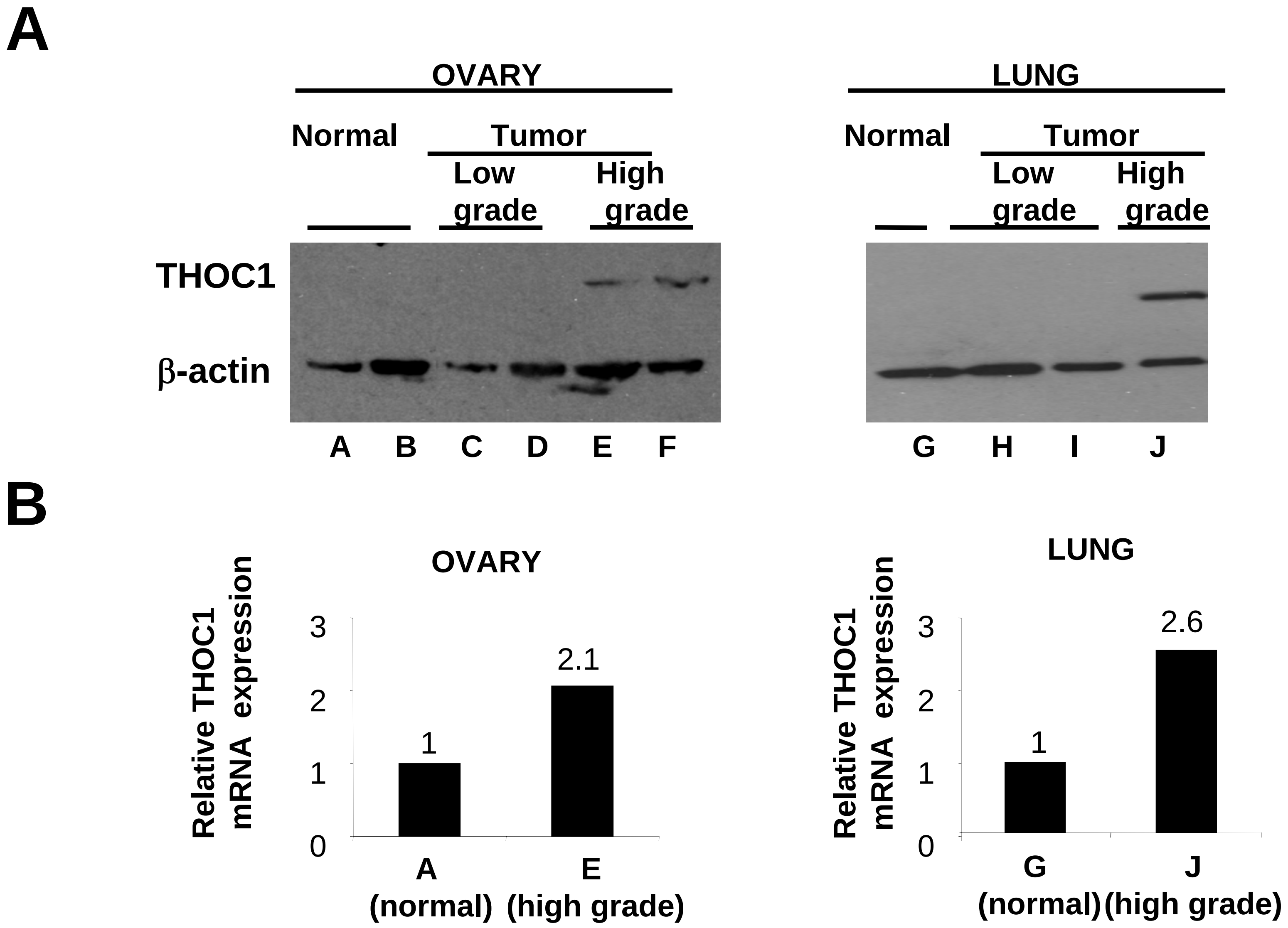

Additional file 4. THOC1 expresion in Ovary and Lung tumors. A) Western blot of THOC1 and β-actin (as endogenous control) in Ovary and Lung tissues. B) THOC1 mRNA relative expression in ovary and lung tissues measured by RT-PCR.
